# Global research alliance in infectious disease: a collaborative effort to combat infectious diseases through dissemination of portable sequencing

**DOI:** 10.1186/s13104-022-05927-2

**Published:** 2022-02-12

**Authors:** Lucky R. Runtuwene, Nuankanya Sathirapongsasuti, Raweewan Srisawat, Narumon Komalamisra, Josef S. B. Tuda, Arthur E. Mongan, Gabriel O. Aboge, Victoria Shabardina, Wojciech Makalowski, Dela Ria Nesti, Wayan T. Artama, Lan Anh Nguyen-Thi, Kiew-Lian Wan, Byoung-Kuk Na, William Hall, Arnab Pain, Yuki Eshita, Ryuichiro Maeda, Junya Yamagishi, Yutaka Suzuki

**Affiliations:** 1 Division 1, AIDS Research Center, National Institute of Infectious Diseases, Tokyo, Japan; 2 Department of Computational Biology and Medical Science, Graduate School of Frontier Sciences, The University of Tokyo, Kashiwa, Japan; 3 Section of Translational Medicine, Faculty of Medicine, Ramathibodi Hospital, Mahidol University, Bangkok, Thailand; 4 Department of Medical Entomology, Faculty of Tropical Medicine, Mahidol University, Bangkok, Thailand; 5 Faculty of Medicine, Sam Ratulangi University, Manado, Indonesia; 6 Department of Public Health, Faculty of Veterinary Medicine, University of Nairobi, Nairobi, Kenya; 7 Institute of Evolutionary Biology, CSIC-Universitat Pompeu Fabra, Barcelona, Spain; 8 Institute of Bioinformatics, Faculty of Medicine, University of Muenster, Muenster, Germany; 9 Department of Bioresources Technology and Veterinary, Vocational College, Universitas Gadjah Mada, Yogyakarta, Indonesia; 10 Department of Biochemistry, Faculty of Veterinary Medicine, Universitas Gadjah Mada, Yogyakarta, Indonesia; 11 Center of Biomedical Research, National Institute of Hygiene and Epidemiology, Hanoi, Vietnam; 12 Department of Biological Sciences and Biotechnology, Faculty of Science and Technology, Universiti Kebangsaan Malaysia, Kuala Lumpur, Malaysia; 13 Department of Parasitology and Tropical Medicine, College of Medicine, Gyeongsang National University, Jinju, South Korea; 14 Centre for Research in Infectious Diseases, University College Dublin, Dublin, Ireland; 15 Pathogen Genomics Laboratory, Biological and Environmental Sciences and Engineering (BESE) Division, King Abdullah University of Science and Technology, Thuwal, Saudi Arabia; 16 Division of Collaboration and Education, International Institute for Zoonosis Control, Hokkaido University, Sapporo, Japan; 17 Division of Biomedical Science, Department of Basic Veterinary Medicine, Obihiro University of Agriculture and Veterinary, Obihiro, Japan

**Keywords:** International collaboration, Portable sequencing, Field sequencing, MinION

## Abstract

**Objective:**

To disseminate the portable sequencer MinION in developing countries for the main purpose of battling infectious diseases, we found a consortium called Global Research Alliance in Infectious Diseases (GRAID). By holding and inviting researchers both from developed and developing countries, we aim to train the participants with MinION’s operations and foster a collaboration in infectious diseases researches. As a real-life example in which resources are limited, we describe here a result from a training course, a metagenomics analysis from two blood samples collected from a routine cattle surveillance in Kulan Progo District, Yogyakarta Province, Indonesia in 2019.

**Results:**

One of the samples was successfully sequenced with enough sequencing yield for further analysis. After depleting the reads mapped to host DNA, the remaining reads were shown to map to *Theileria orientalis* using BLAST and OneCodex. Although the reads were also mapped to *Clostridium botulinum,* those were found to be artifacts derived from the cow genome. An effort to construct a consensus sequence was successful using a reference-based approach with Pomoxis. Hence, we concluded that the asymptomatic cow might be infected with *T. orientalis* and showed the usefulness of sequencing technology, specifically the MinION platform, in a developing country.

**Supplementary Information:**

The online version contains supplementary material available at 10.1186/s13104-022-05927-2.

## Introduction

Infectious diseases are still a threat to the majority of people living in developing countries. According to the World Health Organization, infectious diseases are one of the major causes of death in low-income countries [1]. These diseases are usually caused by bacteria, viruses, parasites, or fungi, and manifest in many forms as a result of injuries caused to various human organs. Infectious diseases are normally known to be transmitted through direct exposure to either an infected person or by an intermediate animal host. Furthermore, with the advancement of international transportation systems involving the movement of people and animals, many infectious diseases have easily spread worldwide with some of these outbreaks having the potential of becoming a pandemic. To mitigate the harmful effects of infectious diseases, combined collaborative efforts have been undertaken amongst many stakeholders. One such collaborative effort has involved the application of One Health to protect human health by monitoring and controlling the health of animals and the environment [2]. The collaborative One Health approach is interdisciplinary and is spread across the boundaries of many countries involving various government agencies. Focusing on human health, the Indonesian Ministry of Health in collaboration with the U.S. National Institute of Health has constructed an infectious disease research network in Indonesia comprising of hospitals, medical faculties, and research centers across the country called the Indonesia Research Partnership on Infectious Disease (INA-RESPOND), which serves as a national forum for infectious disease research on a national scale [3]. In Japan, the government has facilitated the formation of the Global Research Collaboration for Infectious Disease Preparedness (GLoPID-R) to encourage information exchange between member countries, thereby ensuring preparedness for emerging infectious disease outbreaks [4].

In this research note, we report another collaborative effort involving the Global Research Alliance for Infectious Disease (GRAID) consortium, which is a platform for scientists around the world to contribute to the fight against infectious diseases and its achievements. Presently, the main activities of GRAID involve education and training on how to operate the MinION sequencer. Since its introduction, this portable DNA sequencer has changed the way we do genome research. This sequencing technique has the advantages of low overhead cost, simplicity of library preparation, real-time sequencing, and a small sequencer size as compared to the other sequencing techniques. Data acquired from MinION can be analyzed real-time, encouraging its use in clinical settings where a fast identification of pathogens is needed. The portability of MinION enables it to be transported to the field easily, thereby enabling DNA sequencing in situations where it may be impossible to ship samples to a laboratory. In term of technology advancement, MinION is a long-read sequencer, which is able to sequence up to 1 Mb of nucleotide without interruption. It sequences nucleic acids in their native state, so modifications of DNA and RNA strands can be sought for epigenetic analysis. A good example is the use of the MinION sequencing technique in Africa to diagnose an outbreak of the Ebola virus in 2016. Here, the MinION tool was used to confidently diagnose patients infected with the Ebola virus in Africa [5]. Recently, in 2018, the MinION sequencer was used to show that the circulating Lassa virus is not transmissible to humans, thereby assisting in the management of the outbreak [6]. Although MinION has higher error rate compared to the second generation next-generation sequencers, limiting its use in researches that demand high accuracy, the recent developments in new chemistries and bioinformatics tools allow a higher accuracy rate of raw read up to 99% [7].

Our GRAID initiative opens opportunities for scientists from developing countries to have hands-on experience with sequencing technology. So far, we have held six training courses in four different countries, involving delegates from three continents: Asia, Europe, and Africa. GRAID is committed to becoming the pioneer consortium for introducing portable MinION sequencing for infectious diseases researches in developing countries. So far, we have published research papers within the GRAID framework: a genetic polymorphisms analysis of malaria parasite’s chloroquine resistance genes [8], a comprehensive transcriptome analysis of malaria–host interaction [9], serotyping of dengue virus using MinION [10], and a comprehensive survey of drug resistance in malaria parasites [11]. Additionally, specialized software for easy analysis and interpretation of MinION-generated data has been developed within the consortium [12, 13].

To illustrate the activities of the consortium, we will describe a result from a training course held in Yogyakarta, Indonesia in 2019. The organizer provided cattle’s blood samples collected from Kulon Progo District, Yogyakarta Province, Indonesia as part of routine surveillance of blood pathogens in farm animals. The participants were involved directly in all aspects of the sample preparation and sequencing, starting from DNA extraction, DNA concentration measurement, library preparation, and sequencing. The purpose of the training course was to identify the pathogens in the blood samples using a metagenomic sequencing approach. Participants also performed identification of the infecting pathogens using BLAST and One Codex web services. The whole genome of the pathogen was constructed outside of the training course.

## Main text

### Materials and methods

Two whole blood samples of asymptomatic *Bos taurus* were collected in a survey of blood pathogens in farm animals in 2019. In the training course, the DNA was extracted using an HMW DNA extraction kit (Qiagen) by two groups of participants from 200 µl of whole blood. Library preparation was performed using the Ligation Sequencing Kit (SQK-LSK109, ONT), according to the manufacturer’s protocol. The libraries were then sequenced on two flow cells using two MinION sequencers connected to two laptops. Sequencing was performed for 24 h. Base calling was performed individually using ONT’s Guppy version 3.6.1 + 249406c by the participants using their laptops. FASTQ was depleted of host reads by omitting the reads mapped to the host (*Bos taurus*, accession number: ARS-UCD1.2) using a combination of Minimap2 [14] version 2.17-r941 and in-house scripts. The remaining reads were subjected to BLAST [15] and One Codex [16] processes to identify the pathogens. We also attempted to construct the whole genome of the pathogens using a de novo approach with Flye [17] version 2.5 and a reference-guided approach with ONT’s Pomoxis version 0.3.6 on a separate occasion. Before genome construction, reads correction and adapter trimming were performed with Miniscrub [18] version 0.3.1 and Porechop [19] version 0.2.4, respectively. QUAST [20] was used to visualize the genome assembly. All parameters were default.

### Results

MinION sequencing of two samples performed in the training condition was not optimal. Although one sample yielded a good amount of data, the other one did not (Additional file 1: Table S1). The sample with low data yield did not have enough starting DNA concentration and the concentration was further reduced in the library preparation step. Post-extraction DNA concentrations were 759 ng and 430 ng, which were rather low for sequencing. Following post-library-preparation, DNA concentrations were reduced even further to 232 ng and 73 ng, amounts below ONT’s recommendation. Most of the DNA loss was due to the magnetic beads cleaning process. We noticed that participants left the DNA-bound magnetic beads for a long time in the drying process while doing DNA purification, which might have contributed to a huge DNA loss. Because most of the participants joining our hands-on MinION training course would have never done any library preparations for next-generation sequencing, this should be a point to stress out in future training courses.

**Fig. 1:**
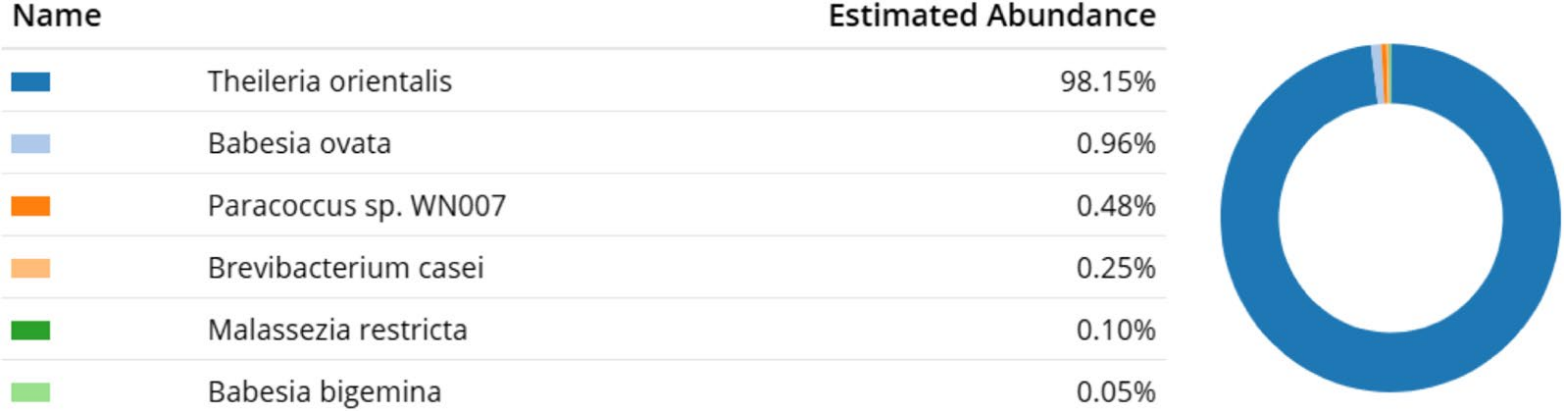
One Codex result of the 3654 remaining reads mapped to its database shows that 98.15% of the reads are in high abundance for *T. orientalis*

Using Minimap2, the participants mapped only the pass reads (249,337 reads) to the *Bos taurus* reference genome (NCBI accession number: ARS-UCD1.2). Pass reads were reads exceeding the average quality score threshold of 7, calculated automatically by the base-calling software. Exactly 245,683 reads (98.53%) were mapped to the cow genome reference and these reads were omitted from further analyses using in-house scripts (Additional file 1: Table S2). To identify the bacteria species, the participants used two web services on the remaining reads: BLAST and One Codex. BLAST results showed that the reads were mostly mapped to *Theileria orientalis* (Additional file 1: Table S3). Approximately 300 reads were mapped to *Clostridium botulinum,* but a re-BLAST of these reads revealed that these reads were also mapped to the reference genome, suggesting a non-specific mapping (Additional file 1: Table S4). On the other hand, reads mapped to *T. orientalis* were assigned with high confidence to the organism (Additional file 1: Table S5). One Codex also showed an agreement with the BLAST results, demonstrating that the *T. orientalis* genome was 98.15% abundant (Fig. 1) in the cow’s blood sample.

**Fig. 2:**
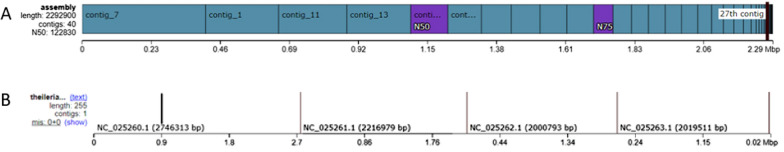
QUAST’s Icarus Genome Viewer results of a genome assembly using the pass reads. The assembly yielded 40 contigs with a total length of 2.29 Mbp. The purple-colored boxes mark the contigs making up for N50 and N75 (**A**). Contig 15 is shown to be mapped to the *T. orientalis* reference genome. The position in the genome is marked by the bold black line (**B**)

Following identification and outside of the training course, we performed construction of the *T. orientalis* genome. Flye software was not able to construct the genome from reads exclusively mapped to *T. orientalis* because of the low number of these reads (Additional file 1: Table S2). Instead, we constructed the genome from all of the pass reads (249,337 reads). The assembly yielded 40 contigs with a total length of 2,292,900 bp, suggesting the assemblies were incomplete for both the host and the pathogens (Fig. 2A). When we mapped the contigs to *T. orientalis* reference genome, only contig 15 was successfully mapped. The contig length was 35,061 bp and only 255 bp was mapped to the reference genome, suggesting non-assembly (Fig. 2B). The identity of this mapping was 95.69%.

**Fig. 3:**
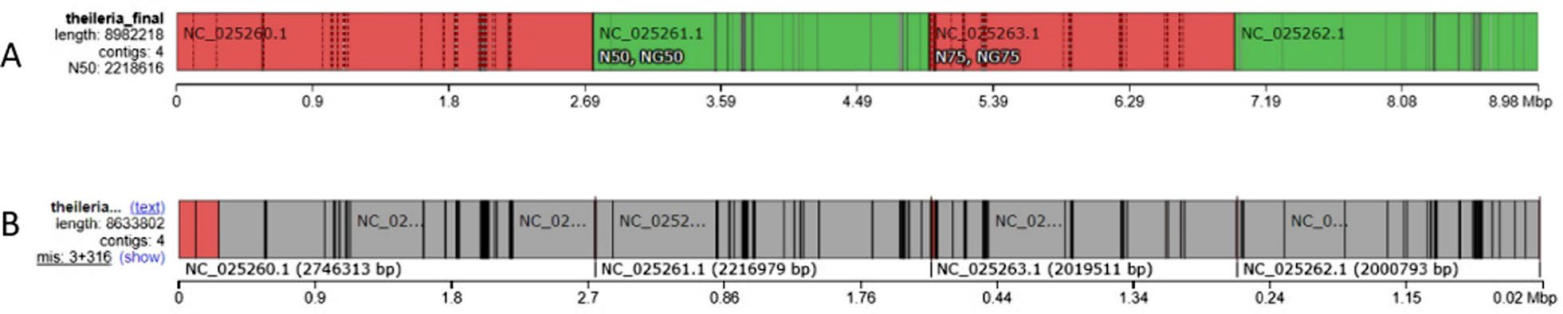
QUAST’s Icarus Genome Viewer results of a genome assembly using reads exclusively mapped to *T. orientalis*, corrected with MiniScrub, and assembled with Pomoxis. Red colored boxes indicate the contigs containing misassembled blocks. The dashed black lines indicate the block boundaries used by Quast to analyze the contigs (**A**). The misassembled blocks and their positions in the genome are shown in details. The black lines indicate the block boundaries (**B**)

As an alternative, we tried a reference-guided construction using Pomoxis. After chopping the reads exclusively mapped to *T. orientalis* with Porechop to remove the adapters and correcting the reads with Miniscrub, we were able to construct 95.78% of the parasite genome. The assembly yielded four contigs, with the largest contig being 2,746,726 bp. Each contig corresponded to each of the parasite’s chromosome. There were three misassembled blocks contained in two contigs (colored red in Fig. 3A). The position of the blocks within the genome is shown in Fig. 3B. Although the assembly was not optimal due to the low reads number, the overall results strongly suggested a real presence of the pathogen in an asymptomatic host.

### Conclusion

This research note has introduced GRAID, an international consortium focusing on training and application of genome research on infectious diseases. Within the GRAID framework, we expect to disseminate the portable sequencing to improve genome researches in developing countries. All of our activities can be monitored via our website: http://bioinformatics.uni-muenster.de/graid/index.hbi. The website provides not only information on our activities but is also a central point for the exchange of information on lab protocols, related to this endeavor. We believe, with our activities that focus on portable sequencer MinION, we will be able to broaden this technology to countries where sequencing is still not a routine research technology. This will eventually open new exciting studies, such as identifying disease-causing agents in an outbreak where shipping samples to another center or country is not possible. As an example, we presented a result of a metagenome analysis from a cow’s blood. The presence of *T. Orientalis* genome found in a routine survey is crucial because the parasite is benign and the infection is mainly asymptomatic. However, a *T. Orientalis* infection is economically important, because non-infected animals introduced into an endemic area or animals under stress could get infected [21]. The *T. Orientalis* strain Ikeda has caused major economic losses in Asia, New Zealand, and Australia, primarily as a result of death or illness in beef and dairy cattle and ongoing milk losses [22]. An analysis of one dairy affected by *T. Orientalis* in New Zealand in 2014 estimated a loss of more than $400 per cow [23]. This can be prevented by comprehensively analyzing pathogens in a routine survey which can be simplified by sequencing technology and GRAID will work towards the dissemination of this technology, specifically the MinION platform.

### Limitations

We described the results of a sequencing obtained from one of our training courses. Because of the untrained participants, only one of the samples yielded enough sequencing reads for analysis. For the sample with enough sequencing yield for identification, we could not generate de novo whole-genome assembly because of the limited number of pathogens’ reads. The recent introduction of Nanopore Adaptive Sampling to remove the host DNA on the go may be beneficial and will be introduced into our next training courses.

## Supplementary Information

**Additional file 1:** **Table S1.** Statistics of the metagenome sequencing process. **Table S2.** Number of reads used for downstream analysis. **Table S3.** The top ten of the BLAST results. **Table S4.** The top ten of the BLAST results of the reads mapped to *Clostridium* *botulinum.* **Table S5.** The top ten of the BLAST results of the reads mapped to *Theileria orientalis.*

## Data Availability

Sequencing data is available in DNA Data Bank of Japan (DDBJ) under accession number DRA012928.

## Abbreviations

BLAST: Basic Local Alignment Search Tools
HMW DNA: High molecular weight deoxyribonucleic acid
NCBI: National Center for Biotechnology Information
ONT: Oxford Nanopore Technologies
