# Supplementary material for: Global research alliance in infectious disease: a collaborative effort to combat infectious diseases through dissemination of portable sequencing
Source: BMC Res Notes. 2022 Feb 12;15:44. doi: 10.1186/s13104-022-05927-2 (PMC8840504; doi:10.1186/s13104-022-05927-2)
Supplement: Supplementary file 1 — Additional file 1: Table S1. Statistics of the metagenome sequencing process. Table S2. Number of reads used for downstream analysis. Table S3. The top ten of the BLAST results. Table S4. The top ten of the BLAST results of the reads mapped to Clostridium botulinum. Table S5. The top ten of the BLAST results of the reads mapped to Theileria orientalis. [file 13104_2022_5927_MOESM1_ESM.docx]

**Additional file 1: Materials**

Table S1. Statistics of the metagenome sequencing process.

|  | 311019_unknown | 44_Jogja |
| --- | --- | --- |
| Total yield in Gb  Total reads  N50 length  Median length  Max length  Median q value | 3.93  347,680  25,206  5,522  184,990  8.2 | 0.48  51,829  22,559  4,283  453,524  8.3 |

Table S2. Number of reads used for downstream analysis.

| Total number of pass reads | Number of reads mapped to *Bos Taurus* (%) | Number of reads used for downstream analysis | |
| --- | --- | --- | --- |
|  |  | Non-specific (%) | Mapped to *Theileria orientalis* (%) |
| 249,337 | 245,683 (98.53%) | 2,617 (1.05%) | 1,037 (0.42%) |

Table S3. The top ten of the BLAST results revealed that the majority of the reads are mapped to *Theileria orientalis* and *Clostridium botulinum*.

| Reads number | NCBI accession number | Description |
| --- | --- | --- |
| 316 | AP011946 | Theileria orientalis strain Shintoku DNA, chromosome 1, complete genome |
| 314 | CP027778 | Clostridium botulinum strain Mfbjulcb6 chromosome, complete genome |
| 256 | AP011947 | Theileria orientalis strain Shintoku DNA, chromosome 2, complete genome |
| 216 | AP011949 | Theileria orientalis strain Shintoku DNA, chromosome 4, complete genome |
| 215 | AP011948 | Theileria orientalis strain Shintoku DNA, chromosome 3, complete genome |
| 45 | CP033842 | Cutibacterium acnes strain FDAARGOS_503 chromosome, complete genome |
| 44 | V00122 | bovine satellite DNA fragment |
| 40 | CP027096 | Bos mutus isolate yakQH1 chromosome 28 |
| 39 | CP027089 | Bos mutus isolate yakQH1 chromosome 21 |
| 37 | CP027082 | Bos mutus isolate yakQH1 chromosome 14 |

Table S4. The top ten of the BLAST results of the reads mapped to *Clostridium botulinum* reveal that the reads are also mapped to the host genome, suggesting non-specific mapping.

| Reads number | NCBI accession number | Description |
| --- | --- | --- |
| 314 | CP027778 | Clostridium botulinum strain Mfbjulcb6 chromosome, complete genome |
| 24 | V00115 | Bos taurus 1.711a satellite DNA with insert INS-1.711A. (extends from base 501 to 1151) |
| 21 | KX592814 | Bos taurus isolate Dominette_000065F genomic sequence |
| 17 | CP027093 | Bos mutus isolate yakQH1 chromosome 25 |
| 17 | LK054987 | Babesia bigemina genome assembly Bbig001, scaffold Bbigscaff_63091 |
| 17 | V00122 | bovine satellite DNA fragment |
| 12 | CP027096 | Bos mutus isolate yakQH1 chromosome 28 |
| 11 | CP027095 | Bos mutus isolate yakQH1 chromosome 27 |
| 9 | CP027085 | Bos mutus isolate yakQH1 chromosome 17 |
| 9 | CP027092 | Bos mutus isolate yakQH1 chromosome 24 |

Table S5. The top ten of the BLAST results of the reads mapped to *Theileria orientalis* reveal that the reads are confidently mapped to the organism.

| Reads number | NCBI accession number | Description |
| --- | --- | --- |
| 316 | AP011946 | Theileria orientalis strain Shintoku DNA, chromosome 1, complete genome |
| 256 | AP011947 | Theileria orientalis strain Shintoku DNA, chromosome 2, complete genome |
| 215 | AP011948 | Theileria orientalis strain Shintoku DNA, chromosome 3, complete genome |
| 216 | AP011949 | Theileria orientalis strain Shintoku DNA, chromosome 4, complete genome |
| 1 | D12691 | Theileria sergenti mRNA for Ts-32K protein, complete cds |
| 1 | XM_009690545 | Theileria orientalis strain Shintoku ABC transporter partial mRNA |
| 1 | XM_009690642 | Theileria orientalis strain Shintoku uncharacterized protein partial mRNA |
| 1 | XM_009691017 | Theileria orientalis strain Shintoku conserved hypothetical protein partial |
| 1 | XM_009691157 | Theileria orientalis strain Shintoku ABC transporter partial mRNA |
| 1 | XM_009691311 | Theileria orientalis strain Shintoku ubiquitination-mediated degradation co |
